# Computed tomography for myocardial characterization in ischemic heart disease: a state-of-the-art review

**DOI:** 10.1186/s41747-020-00158-1

**Published:** 2020-06-17

**Authors:** M. van Assen, M. Vonder, G. J. Pelgrim, P. L. Von Knebel Doeberitz, R. Vliegenthart

**Affiliations:** 1 University Medical Center Groningen, University of Groningen, Hanzeplein 1, 9713 EZ, Groningen, The Netherlands; 2 Department of Epidemiology, University Medical Center Groningen, University of Groningen, Groningen, The Netherlands; 3 Department of Radiology, University Medical Center Groningen, University of Groningen, Groningen, The Netherlands; 4 Institute of Clinical Radiology and Nuclear Medicine, University Medical Center Mannheim, Medical Faculty Mannheim, Heidelberg University, Mannheim, Germany

**Keywords:** Tomography, x-ray computed, Myocardial ischemia, Myocardial infarction, Myocardial perfusion imaging, Late iodine enhancement

## Abstract

This review provides an overview of the currently available computed tomography (CT) techniques for myocardial tissue characterization in ischemic heart disease, including CT perfusion and late iodine enhancement. CT myocardial perfusion imaging can be performed with static and dynamic protocols for the detection of ischemia and infarction using either single- or dual-energy CT modes. Late iodine enhancement may be used for the analysis of myocardial infarction. The accuracy of these CT techniques is highly dependent on the imaging protocol, including acquisition timing and contrast administration. Additionally, the options for qualitative and quantitative analysis and the accuracy of each technique are discussed.

## Key points

- Myocardial perfusion defects can be detected using high-end CT systems.
- Static myocardial CT perfusion imaging (CT-MPI) allows for low-dose evaluation of myocardial ischemia with good accuracy.
- Dynamic CT-MPI allows for the quantification of myocardial perfusion parameters at reasonable radiation dose (5–9 mSv).
- Late iodine enhancement scanning shows potential for myocardial viability assessment.

## Introduction

The hemodynamic consequences of coronary artery disease (CAD) on the myocardium can be performed using several different approaches. One of these approaches is myocardial perfusion imaging (MPI). Traditionally, MPI is performed by positron emission tomography (PET) or single-photon emission computed tomography (SPECT) or more recently by MRI. These modalities are currently not able to perform anatomical evaluations of the coronary arteries. Combining anatomical and functional evaluation [1] using computed tomography (CT) would be ideal, since it offers the possibility of complete CAD evaluation using one modality. Myocardial CT perfusion imaging (CT-MPI) holds potential for functional evaluation due to recent technical developments. There are two main CT approaches to visualise and quantify myocardial perfusion, static and dynamic CT-MPI [2–4].

Without intervention, impairments of myocardial perfusion will inevitably lead to myocardial damage. Whereas perfusion imaging is mostly focused on finding ischemia and infarction, late enhancement imaging is focused on infarct detection specifically [3]. Late enhancement imaging is mainly used in MRI, and this is currently the reference standard for myocardial infarct (MI) evaluation. However, recent research has shown that CT using iodinated contrast also enables myocardial viability evaluation.

The main purpose of this article is to review the different options for functional cardiac CT analysis for myocardial ischemia and infarction and to provide an overview of presently available techniques.

## Myocardial perfusion imaging

**Fig. 1:**
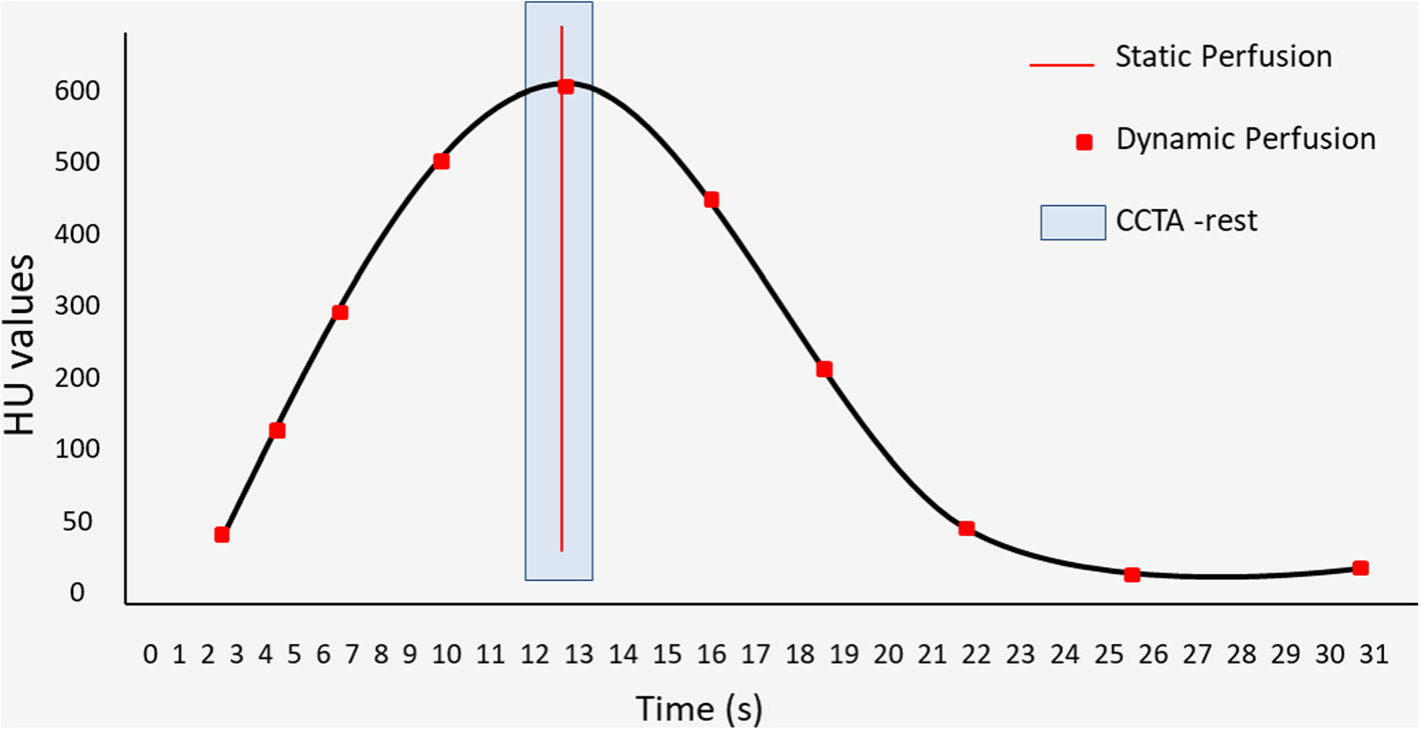
Schematic representation of the imaging time points for static (red line) and dynamic (red dots) perfusion imaging. Each capturing a specific part of the contrast enhancement curves measured in both the aorta (arterial input function) and myocardial tissue (tissue attenuation curve). Both techniques can be acquired at rest or stress. The blue box represents the optimal time window for coronary CT angiography imaging during rest

There are currently two main CT techniques to perform MPI: static and dynamic MPI. Static MPI consists of a single electrocardiogram (ECG)-gated, iodinated contrast-enhanced CT acquisition of the heart. Static CT-MPI can be performed using either single- or dual-energy (SECT or DECT) settings. First described in 2008 [5], DECT is a relatively new technological development in the field of CT imaging with different vendor-specific solutions allowing the quantification of iodine concentrations. Static CT-MPI imaging indirectly evaluates myocardial perfusion by looking at the distribution of contrast (in the case of SECT) or iodine concentrations (in the case of DECT) throughout the myocardium at a single time point. In contrast with static CT-MPI, dynamic CT-MPI acquires multiple images over a specific time period, visualizing the entire in- and outflow of contrast medium. This dynamic nature enables direct quantification of myocardial perfusion. Figure 1 shows the differences between static and dynamic CTMPI.

PET-MPI is currently the reference standard for absolute quantification of myocardial perfusion and SPECT for visual analysis [6]. However, CT in addition offers anatomical CAD evaluation due to a higher spatial resolution at reduced cost [6]. CT is able to directly quantify the myocardial perfusion due to the linear relationship between iodine concentration and the change in signal intensity (HU values) over a wide range of iodine concentrations. This is in contrast to MRI-MPI in which the relationship between gadolinium and signal enhancement [7] becomes non-linear at the gadolinium concentrations that are commonly used for visual MPI analysis [7].

### Technical information and requirements

The most important requirement for CT-MPI is a suitable CT system. For static CT-MPI, a 64-multidetector CT system is the minimum requirement, although wider volume CT systems are preferred. Smaller detector range systems require multiple slabs to image the heart completely which leads to a relatively low temporal resolution, thereby increasing overall scanning time. This increased scanning time can lead to changes in contrast enhancement of the myocardium during the acquisition resulting in both false-positive and false-negative results. The first DECT systems required a decrease in temporal resolution for cardiac acquisitions [8]. However, newer DECT systems addressed this limitation leading to temporal resolutions similar to SECT systems 83 ms or lower, depending on the dual-energy approach [8].

**Fig. 2:**
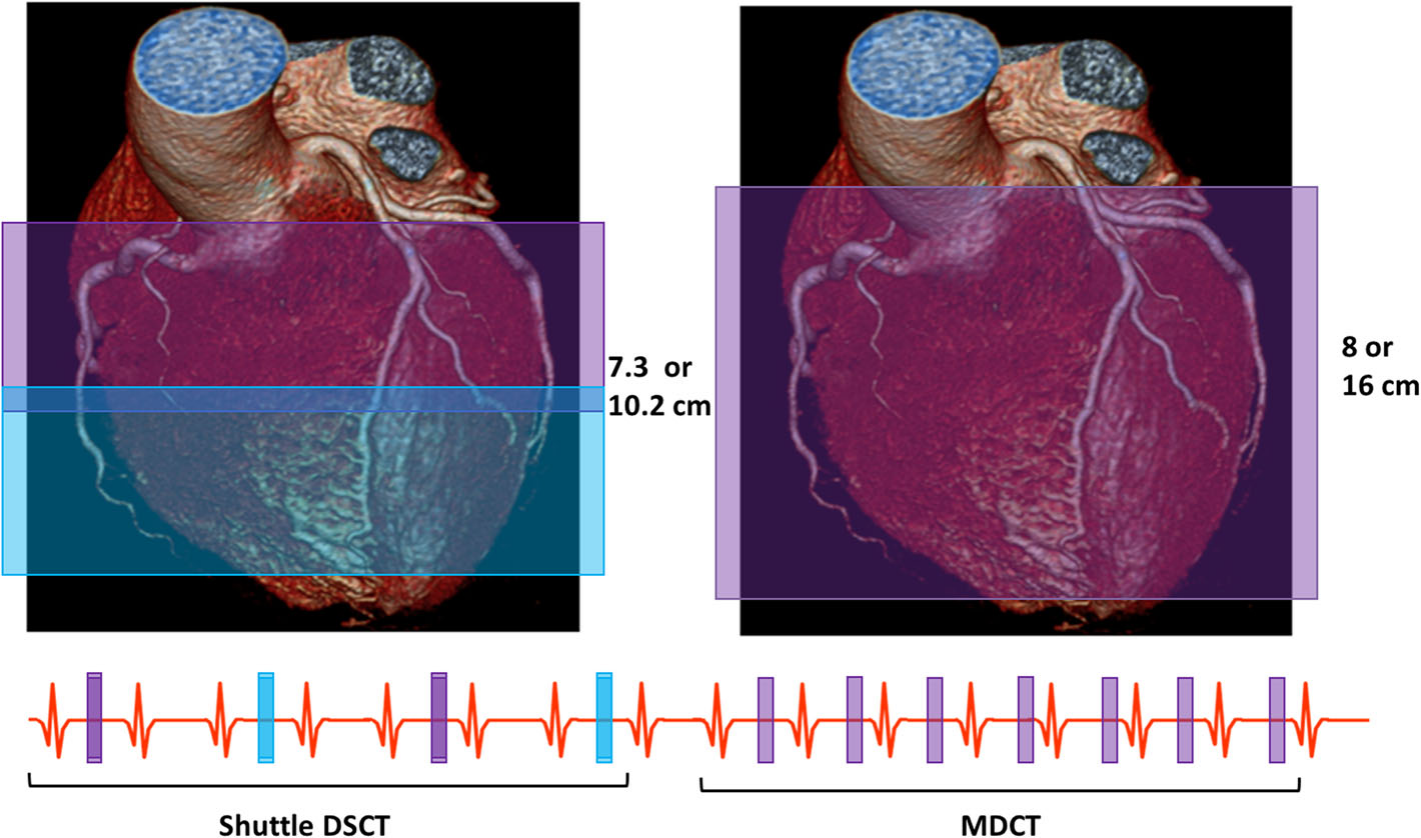
Dual-source CT uses a shuttle mode, alternating table position using two acquisitions merged into one image, to get a *z*-axis of 7.3 or 10.2 cm for the second- and third-generation systems. Multi-detector CT systems use wide detector with 280 or 320 rows to reach a *z*-axis range of 8 or 16 cm, respectively. Both approaches allow for whole-heart imaging

Dynamic CT-MPI is most commonly performed using dual-source CT (DSCT and wide multi-detector CT (MDCT) systems (256–320 rows). Second- and third-generation DSCT scanners, characterised by their relatively high temporal resolution (75 or 66 ms), have a coverage sof 7.3 and 10.2 cm. Using a shuttle mode, consisting of alternating back-and-forth table movements with sequential scanning, allows visualization of most of the left ventricle [8–10]. MDCT systems, used in only a limited number of dynamic CT-MPI studies [11, 12], allow for full-heart coverage within one gantry rotation using a single-tube wide detector with 256 or 320 detector rows covering 8 and 16 cm. The disadvantage of these MDCT systems is the decreased temporal resolution (140 ms), which can result in an increase in motion artifacts and an overall decrease in image quality [8]. However, wide-detector MDCT systems are able to acquire an image every heartbeat in contrast to requiring images every other heartbeat such as with DSCT systems. This is made possible by eliminating the time needed for the shuttle mode. The increase in acquired images will increase the information about the in- and outflow of contrast; however, every additional image comes at a cost of increased radiation dose. Figure 2 shows the difference between the DSCT shuttle mode and the MDCT wide detector approach.

### Radiation dose

Static CT-MPI can be performed at the lowest radiation dose of all CT perfusion techniques, similar to CCTA imaging, with reported radiation doses between 2 and 9 mSv for combined rest-stress examinations [2, 4, 13]. The radiation dose of dynamic CT-MPI is relatively high due to the increased number of acquisitions that need to be taken, resulting in effective dose between 5 and 13 mSv [13, 14]. Nevertheless, recent studies that used the newest high-end CT systems generally show lower radiation doses (5–9 mSv) [11, 15]. Dynamic CT-MPI radiation doses could be reduced by applying new acquisition techniques that require only a limited number of optimally timed acquisitions. Additionally, these timed acquisitions could be used for conventional CCTA evaluation [16].

Comparing SECT with DECT CT-MPI shows that used radiation doses are similar [17–19]. Studies about dual-source DECT imaging show strong evidence that DECT imaging is not associated with increased radiation dose levels compared to SECT acquisitions acquired at 120 kVp [20]. Detector-based DECT systems are expected to produce similar radiation doses to SECT. However, these detector-based DECT scans are standardly acquired at a high tube voltage, and therefore, the tube current should be lowered to maintain similar radiation dose levels [19].

Traditionally, radiation exposure can be reduced by using lower kVp levels (100 kVp instead of 120 kVp) while maintaining optimal image quality in high-end systems. Nonetheless, DECT scanning at lower kVp will only marginally reduce the radiation dose compared to SECT, since both low and high kVp spectra are fundamentally required for DECT. In addition, dual-source DECT systems offer the use of a tin filter (Sn). Sn filters are used to block low-energy photons of the high-energy beam (100/150 kVp) improving spectral separation and reducing total radiation dose [21].

### Image protocol

#### Exam preparations

Patients should be instructed to not consume any caffeine-containing substances (coffee, tea, chocolate, etc.) for 24 h before the examination, because of the interfering effects of caffeine on the effectiveness of the stressor agent. Especially adenosine is sensitive to the competing effect of caffeine, while early-stage research indicates that regadenoson is less affected [22, 23].

Heart rate-limiting medication such as beta-blockers and coronary dilating medication such as sublingual nitrates are known to influence myocardial perfusion; therefore, it is advised to only use them when necessary to achieve diagnostic image quality for CCTA imaging [24, 25].

#### CCTA, rest, and stress phase acquisitions

Traditionally, MPI can be performed by using a rest and stress phase acquisition to discriminate myocardial ischemia from infarction. Ischemic defects are present on stress images only while infarcted defects are present on both rest and stress images. Using CT, these functional acquisitions can be performed in conjunction with a CCTA acquisition for anatomical coronary evaluation.

For static CT-MPI, the rest phase acquisition is similar to the CCTA and can be performed with the standard CCTA protocol [3]. For dynamic CT-MPI, the CCTA need to be acquired separately due to the dynamic nature of the rest acquisition. For dynamic CT-MPI, the use of CCTA, rest, and stress acquisitions will result in high radiation doses. Therefore, a stress-only approach is used in several dynamic CT-MPI studies [9, 26]. By using the quantification abilities of dynamic CT-MPI, discrimination between ischemic and infarcted myocardium is still possible.

**Fig. 3:**
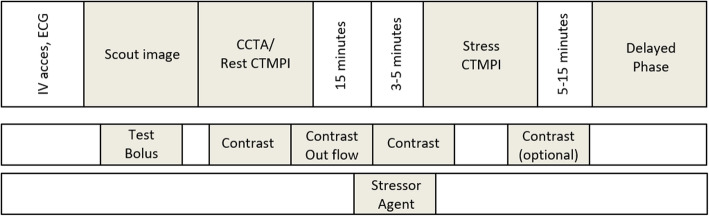
Schematic representation of cardiac imaging protocol, including anatomical evaluation using coronary CT angiography (or rest perfusion in case of static perfusion imaging) and functional evaluation using rest/stress CT-myocardial perfusion imaging. An optional acquisition can be used for the detection of myocardial scarring using delayed phase imaging

Point of debate is the order of acquisitions, which will ultimately depend on which evaluation takes precedence. In patients with a high probability or known CAD or in patients with stents or bypasses, myocardial ischemia is likely, and functional evaluation is more important than anatomical evaluation. In these cases, stress CT-MPI imaging should be performed first, eliminating the effect of contrast contamination and the suppressing effect of beta-blockers and nitroglycerin on myocardial ischemia [24, 25]. In patients with a low to intermediate probability of CAD, it would be preferable to start with CCTA (rest phase for static CT-MPI), in order to avoid unneeded testing in case of no stenosis. An important factor for successful multi-acquisition imaging is to allow sufficient time between each examination to allow complete outflow of contrast agent to avoid contrast contamination and reduce the effect the stressor agent for the subsequent acquisition. Figure 3 shows a schematic representation of a complete cardiac work-up protocol, including CCTA, perfusion, and delayed enhancement imaging.

#### Stressor agents

For stress CT-MPI, a pharmacological stressor agent is used to achieve maximal hyperemia, with adenosine the most frequently used. However, the use of adenosine causes unwanted short-term side effects such as bronchial constriction. This is especially apparent in patients with reactive airway disease, such as COPD, a frequent comorbidity in patients with CAD. Although the short half-life of adenosine allows for the abrupt discontinuation of administration and rapid disappearance of potential harmful side effects, it requires continuous intravenous administration during acquisition and weight-based dosing [23, 27, 28]. Another stressor agent that is increasingly used is regadenoson. It is a potent and selective coronary vasodilator with a rapid onset of action and a longer half-life compared to adenosine. Therefore, regadenoson can be administered as a fixed-dose bolus without weight adjustments. Furthermore, given its selectivity, there are less serious side effects making it also suitable and safe to use in COPD patients [22, 29]. Nevertheless, some studies also report negative effects of the use regadenoson, like higher rates of arrhythmias and increased use of rescue agent aminophylline [30]. Concluding, stress acquisitions require the administration of adenosine for 2–5 min with a rate of 140 μg/min/kg or a single injection of 0.4 mg of regadenoson.

#### Acquisition timing

For static CT-MPI, scan timing is an important factor. The image should be acquired during the early arterial phase of first-pass contrast enhancement at the peak of the contrast enhancement curve. The timing of the peak can be estimated using a test-bolus or bolus-tracking technique. Optimal time delays vary between 2 and 4 s depending on measurement location (ascending or descending aorta) and on the HU threshold (150 or 250 HU) [31].

For dynamic CT-MPI, temporal sampling rates become a significant factor. Several studies have shown that the limited temporal sampling rates that are currently being used lead to an underestimation of the perfusion compared to the true perfusion values and PET determined values [32, 33].

Another important timing factor for dynamic CT-MPI is the timing of the images with respect to the cardiac phase. Myocardial perfusion is not constant but varies between the systolic and diastolic phase [34, 35]. As a result of the myocardial contraction, myocardial perfusion takes place during the diastolic phase and is maximal end-diastolic. However, systolic phase imaging offers some important advantages. Firstly, during systole, the heart is contracted, resulting in a smaller heart volume and in particular a shorter basal-apical length. This is beneficial in scanner systems with limited heart coverage. Wide-detector MDCT studies are performed mostly in diastolic phase, while still being able to capture the entire heart at its maximal volume [11, 36]. Secondly, the systolic phase is constant in duration independent of heart rate and is less sensitive to arrhythmia which is beneficial during stress acquisitions. Finally, because of the reduced volume and reduced contrast dose in the left ventricle, beam-hardening artifacts are reduced.

### Image analysis

#### Qualitative analysis

CT-MPI can be qualitatively analyzed by visual inspection of the contrast attenuation on short-axis view images of the left ventricle. For optimal assessment of perfusion defects, thick sections, minimum intensity projection, and a narrow window width can be used [37]. A standard AHA-17-segment model is used to describe perfusion defects.

Additionally, for DECT static CT-MPI, virtual mono-energetic images (VMI) can be created. VMI images offer the advantages of being able to be reconstructed at multiple energy levels by using different percentages of the low and high energy data. Low energy reconstruction (*e.g.,* below 80 kV), will lead to an increase in contrast attenuation exceeding the increase in noise level. These images can be used to assess patients who cannot receive the normal dose of contrast agent or when the contrast-bolus given was sub-optimal [38, 39]. High energy reconstructions (*e.g.,* 110 kV and higher) can be used to reduce blooming artifacts or metal artifacts, enhancing the ability to analyze severe calcified plaques or stents [40, 41].

**Fig. 4:**
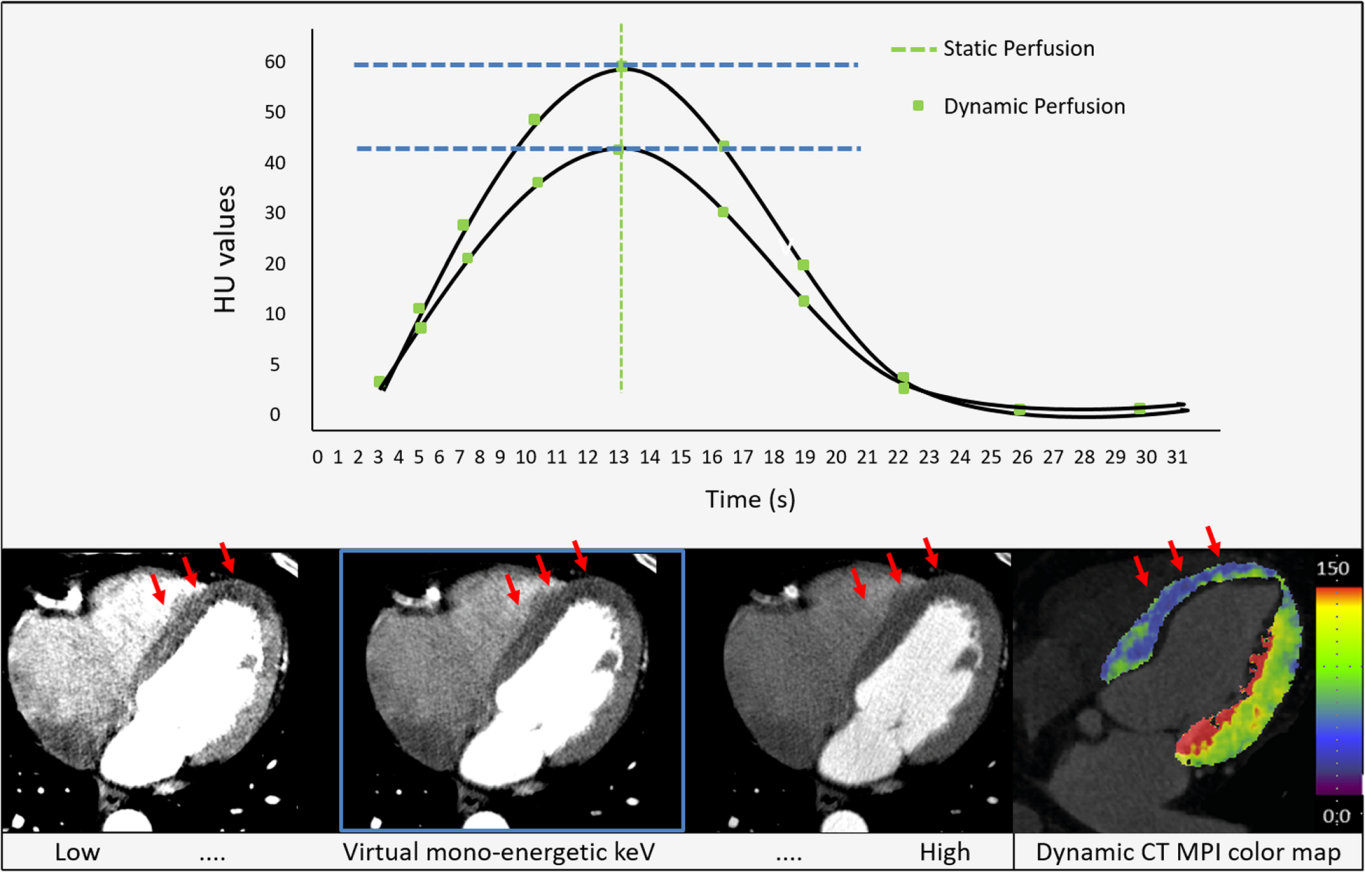
The tissue attenuation curve (top figure) is lower for ischemic areas than for normal areas of the myocardium, causing ischemic myocardium to be visible as hypo-attenuated areas (red arrows, bottom figures). The static CT-MPI images represent multiple kV levels, showing the possibilities of dual-energy CT to enhance the hypo-attenuation of the ischemic area by using varying kV levels exploiting the dual-energy abilities. The blue boxed image represents the traditional single-energy static CT-MPI acquisition. The most right image shows a dynamic CT-MPI color-coded map (mL/100 mL/min) of the short axis based on quantitative myocardial blood flow data

Dynamic CT-MPIMPI offers the possibility of visually analyzing the differences in HU values over time. Regularly, this is represented as a color-coded perfusion maps based on quantitative MBF values, representing MBF values per pixel over time. This approach allows for the visual detection of three-vessel disease. Figure 4 shows an overview of all the options for qualitative analysis

#### Semi-quantitative analysis

Although semi-quantitative analysis of static CT-MPI acquired at single-energy is attempted using parameters such as the transmural perfusion ratio, visual analysis is still superior [42–44].

**Fig. 5:**
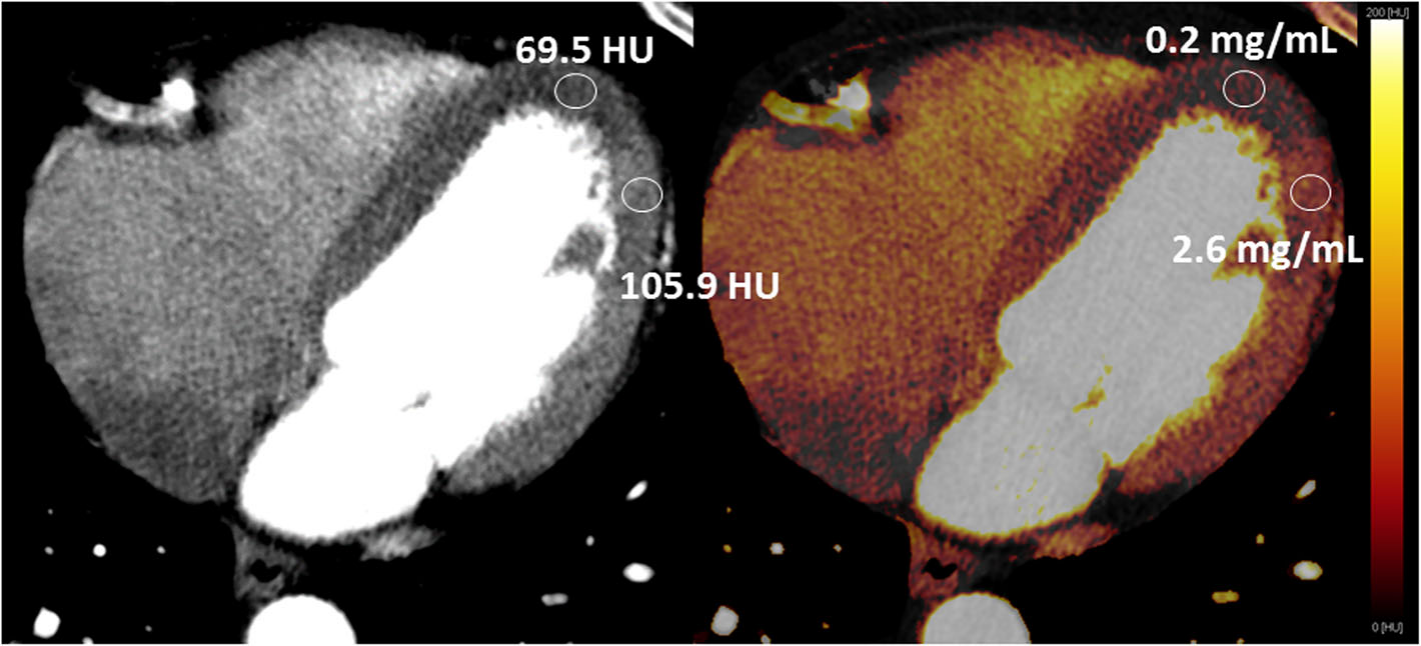
The left image shows a grey scale DECT CCTA image with a clear difference in HU values between ischemic and normal myocardium. The right image represents the iodine map, showing a color-coded map of the iodine concentration, again showing a clear difference in iodine concentration between ischemic and normal myocardium

Currently, only DECT acquisitions allow for semi-quantitative analysis of static CT-MPI using an iodine concentration map, see Fig. 5. The accuracy of the third-generation DSCT and first-generation dual-layer CT (DLCT) systems for the quantification of iodine is shown to be highly accurate [45]. The DSCT systems showed slightly more accurate measurements, especially using the 150Sn/70 or 150Sn/80 kVp combination, with a lower measurement error compared to DLCT systems. Fast kV-switching systems show similarly high accuracy to the DSCT systems [46, 47]. A limited number of studies on iodine quantification have shown that thresholds between 2.1 and 2.5 mg/mL of iodine are optimal to discriminate diseased from normal myocardium using a stress acquisition, while at rest, a threshold of 1.0 mg/mL of iodine can be used to discriminate between infarcted and ischemic myocardium [48, 49].

#### Quantitative analysis

**Fig. 6:**
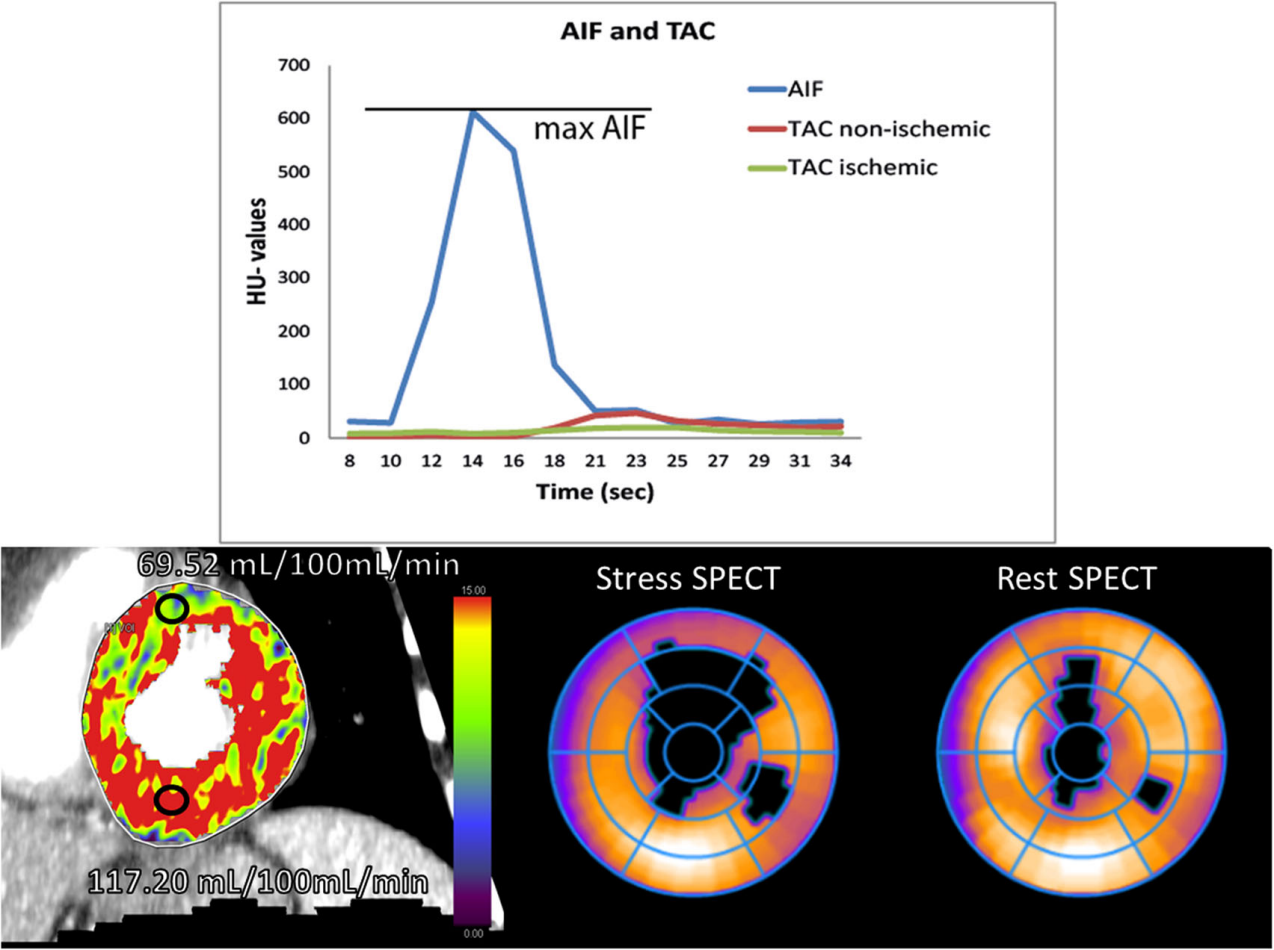
The top image shows a schematic representation of the upslope method used to calculate myocardial blood flow. The lower images show a quantitative perfusion map, based on a deconvolution approach using a two-compartment model combined with the shown upslope method, resulting in quantitative MBF values. A perfusion defect, corresponding to the SPECT perfusion images, is visualised showing significantly lower myocardial blood flow values compared to normal myocardium

Quantitative analysis using dynamic CT-MPI can be performed by direct or indirect methods. Indirect perfusion parameters are derived from the tissue attenuation curves (TAC) and the arterial input function curve (AIF) using the so called upslope method. The upslope method takes the ratio between the maximum upslope of the TAC curve and the maximal AIF value as a measure of perfusion. The main advantage of this method is that it is fairly robust and computationally easy while only the upslope is needed thereby eliminating all acquisitions made after the TAC peak, possibly reducing radiation dose. Figure 6 shows the basic principle of the upslope method based on the attenuation curves.

Direct quantitative analysis of dynamic CT perfusion data is done using a model-dependent deconvolution approach, similar to MRI perfusion [7]. For CT-MPI, there are several models available. From these models, the myocardial blood flow (MBF) can be determined [7, 50, 51]. The most used approach for CT perfusion quantification is a hybrid approach where the deconvolution technique is used to model the curves, and subsequently, the upslope method is used to calculate MBF [52]. Studies have shown that there is no difference in the diagnostic accuracy between different models; however, the absolute values can be significantly different and this should be taken into account when threshold is chosen to detect abnormalities [53]. Figure 6 shows an example of a perfusion map based on the hybrid approach compared to SPECT perfusion imaging results.

##### Absolute *versus* relative perfusion values

A wide variety of absolute and MBF threshold values for the detection of perfusion defects are reported in the literature [13, 14, 54]. Variation in these values can be caused by differences between patient populations, CT systems, image protocols, and analysis procedures. A solution is the use of a relative measure that compares healthy myocardium with ischemic myocardium within a patient. However, using relative values eliminate the benefit of evaluating global perfusion defects.

Several studies reported that relative MBF values yielded a higher diagnostic accuracy compared to absolute MBF values [55–57]. However, one study reported that myocardial flow reserve estimations were not as accurate as individual MBF estimates using PET perfusion as a reference [11].

##### MBF *versus* MBV

Analysis of the myocardial blood volume (MBV) could assist in the discrimination of ischemic and infarcted myocardium in combination with MBF. MBV can be derived by using the peak enhancement of the TAC [58]. In ischemic myocardium, the arterioles dilate to compensate for the decreased flow caused by a coronary stenosis keeping the MBV in the myocardium constant. In infarcted myocardium, this compensatory mechanism is not available leading to a decrease in MBV [58–62].

### Diagnostic accuracy

The largest study on static CT-MPI (381 patients) is the CORE320 study. This study showed an accuracy of 0.93, with a sensitivity and specificity of 80 and 74%, compared to combined SPECT and invasive coronary angiography [63–66]. Another multi-center study (110 patients), performed by Cury et al., investigated the accuracy of static CT-MPI compared to SPECT-MPI and showed slightly higher sensitivity and specificity of 90 and 84% with an overall accuracy of 87% [67]. Meta-analyses on static CT-MPI showed that the average sensitivity and specificity ranged between 75–84% and 78–95% for a variety of image protocols and reference standards used [2, 68].

Overall DECT-MPI studies showed good accuracy compared to various reference modalities [4, 48, 49, 69–73]. Sensitivity and specificity are reported around 89 and 78%. A limited number of studies have been performed on the quantitative analysis of DECT-MPI acquisitions. They showed that a rest acquisition is needed to discriminate between ischemic and infarcted myocardium [48, 49].

For dynamic CT-MPI, similar accuracies have been reported as with static CI MPI with an average sensitivity and specificity ranging between 76–100% and 74–100%, respectively [2, 13, 14, 74, 75]. Quantitative analysis studies have focused on determining an MBF threshold to discriminate ischemic from normal myocardium resulting in a wide range of threshold values between 75 and 136 mL/100 mL/min [13, 14, 54].

## Myocardial viability imaging

Viability imaging, using either MRI or CT, is based on the principle that MRI and CT contrast agents (gadolinium and iodine) accumulate in the intracellular space of the necrotic myocytes. Late iodine enhancement CT (LIE-CT) shares many similarities with late gadolinium enhancement MRI (LGE-MRI), which is the current clinical gold standard for myocardial infarct (MI) detection and viability assessment. In the acute phase of myocardial infarction, the volume of contrast agent is increased due to rupturing of the cell membranes of the necrotic myocytes. In older infarcts, the necrotic cells are replaced by scar tissue consisting out of fibrotic, collagen-rich tissue also leading to an increased volume of contrast agent. This is visualised by hyper-enhanced areas on delayed image acquisitions. LIE-CT hyper-enhancement is not specific for myocardial infarction but rather indicates myocardial tissue with an expanded extracellular matrix of which myocardial infarction is probably the most common one. Other causes of hyper-enhancement are non-ischemic cardiomyopathies, amyloidosis, and sarcoidosis.

### Technical information and requirements

LIE-CT can be performed using two different approaches, SECT or DECT. DECT offers the possibility of material decomposition which allows for the reconstruction of multi-level kV images and quantitative iodine maps. In theory, this will enhance the use of LIE-CT by exploiting the increased iodine enhancement using low-kV images or iodine maps.

DECT evaluation is of particular interest in a patient population with a prevalence of metallic devices since this group of patients is not suitable for MRI evaluation.

### Radiation dose

Radiation doses ranging between 2 and 5 mSv have been reported, mostly using low-dose acquisition protocols utilizing a lower tube voltage [76, 77]. Lowering the tube current has shown not to be as effective as lowering the tube voltage. Although a reduction in tube current reduced the radiation dose, it comes at the cost of an unacceptable degradation of image quality that decreased the accuracy for the detection of MI significantly [77].

Another strategy used to reduce the radiation dose is the use of prospective electrocardiographic (ECG) gating. The use of prospective triggering has shown the ability to significantly reduce radiation dose while maintaining a good agreement with LGE-MRI [77]. Additionally, the scan protocol can be set to a high-pitch mode (available on the second and third dual-source CT systems). This high-pitch mode shows potential in reducing the radiation dose of LIE-CT acquisition achieving radiation doses below 1 mSv while reaching accuracies around 90% compared to LGE-MRI [77].

### Image protocol

#### Contrast agent administration

One of the main variables in LIE-CT imaging protocols is the difference between contrast administration protocols. Currently, there are two main protocols used for LIE-CT, the so-called single-bolus protocol and the bolus-continuous infusion protocol [78]. In the more commonly used bolus protocol, besides the contrast material used for the previous CCTA acquisition, no additional contrast material is injected. In the bolus-continuous protocol, additional contrast is continuously infused (30–90 mL at 0.1–0.3 mL/s) between the CCTA and the LIE-CT acquisition. Continuous infusion is thought to maintain a continuously high intravascular concentration gradient enabling a slow wash-in of contrast agent to the infarcted tissue leading to an increased attenuation difference between infarcted and healthy myocardium [78]. Although there is currently no consensus about which contrast agent administration protocol is superior, the majority of publications on LIE-CT use a single-bolus protocol.

One study showed that the bolus-continuous infusion protocol yielded better image quality than the single-bolus protocol [77]. This increase in image quality was caused by the increased attenuation difference in the infarcted and healthy myocardium, showing a slightly higher correlation with MRI compared to the single-bolus technique [77]. The main disadvantage of the bolus-continuous protocol is the increased LV blood pool attenuation, possibly masking small subendocardial infarcts. Therefore, an additional waiting time of about 5 min between contrast administration and scan acquisition is recommended.

#### Acquisition timing

Another important parameter is the timing of the LIE-CT acquisition in respect to the contrast agent administration. Time intervals of 5–15 min are reported in LIE-CT studies.

Some studies found no difference in protocols using 5–10 min delay, while other studies show that the optimal delay lies somewhere between 3 and 5 min for the single-bolus approach and between 10 and 15 min for the bolus-continuous approach [77, 79]. In order to optimise the LIE-CT workflow, it is important to determine the earliest possible time point that allows for accurate diagnosis taking into account the contrast administration protocol.

### Image analysis

#### Qualitative analysis

**Fig. 7:**
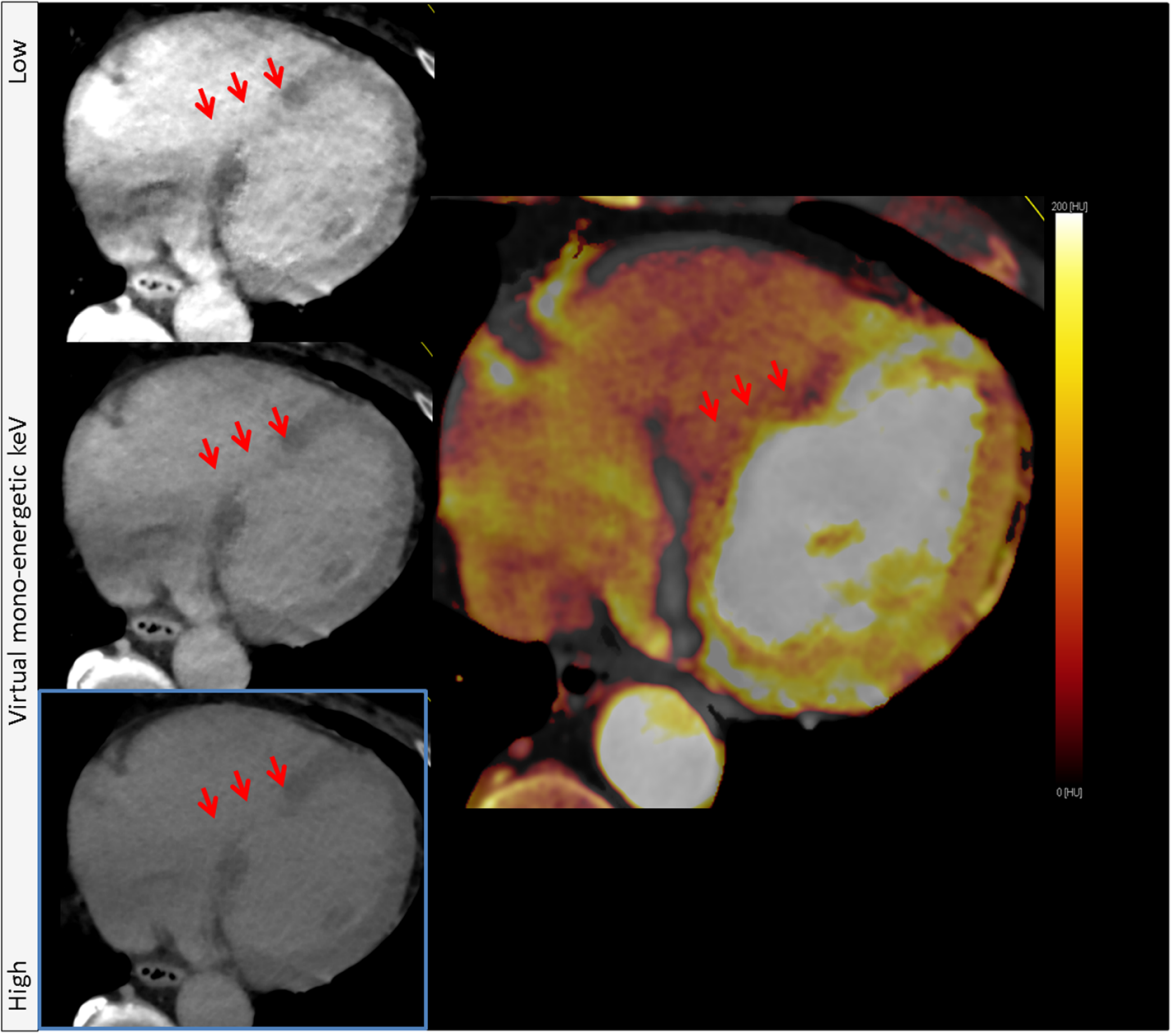
The left images show different level kV LIE-CT images ordered from low to high kV, with the image in the blue box representing the traditional SECT LIE-CT acquisition. The right image represents the iodine map. The red arrows indicate hyper-enhancement of the infarcted areas of the myocardium

As with LGE-MRI, infarcted myocardium can be identified on LIE-CT images by hyper-enhancement. Late enhancement pattern is the most reliable determinant of the underlying disease. It is important to differentiate infarcted from non-infarcted hyper-enhanced areas. DECT acquisition offers some additional advantages for the visual analysis of LIE-CT acquisitions. VMI images or iodine-concentration maps can help enhance specific image features. Studies have demonstrated that low keV images or iodine maps are more suitable for LIE-CT analysis [80, 81], see Fig. 7.

##### Infarct size and enhancement pattern

Several infarct characteristics are important to rapport with regard to prognostication. Studies have shown that larger MI sizes were closely related to myocardial dysfunction and increased the risk for future major adverse cardiovascular events (MACE). Therefore, it is important to report infarct size when analyzing LIE-CT images [82, 83].

Another important characteristic is the enhancement pattern. Infarcted myocardium is characterised by the fact that hyper-enhancement is limited to a specific vascular territory since it is often directly caused by a coronary stenosis. Besides that, infarction always starts at subendocardial part of the myocardium and expands transmurally depending on the severity. A combination of the presence of hyper-enhancement and hypo-enhancement shows better correlation with wall thinning, ejection fraction, microvascular obstruction, and left ventricular remodeling than the presence hyper-enhancement alone and is indicative for future MACE. In a post-PCI study, hypodense areas on LIE-CT images were shown to have a superior prognostic performance for the prediction of MACE [82, 83].

#### Semi-quantitative analysis

##### Transmural extent

Semi-quantitative assessment of the degree of transmurality can aid in the prognostication of patients with myocardial infarction and should therefore be reported. Transmurality can be reported in the percentage of myocardial wall (≤ 25%, 26–50%, 51–75%, and ≥ 76%) involved in the infarction on LIE-CT images for each of the AHA-17 segments. Patients with transmural infarctions show a worse recovery of LV function at 6-month follow-up and were more frequently hospitalised for heart failure compared to patients with subendocardial MI [83–86]. Although infarct transmurality measured on perfusion images shows better correlation with follow-up myocardial dysfunction than transmural LIE-CT enhancement, transmural infarcts show a higher correlation to outcome than subendocardial abnormalities [43].

#### Diagnostic accuracy

Compared to LGE-MRI, LIE-CT demonstrated excellent agreement for the identification of the hyper-enhanced infarcted regions and for infarct size. Sensitivities and specificities around 98% and 94% have been reported [87]. However, only few studies have been performed on this topic and data is lacking. Due to the relatively poor resolution of LIE-CT and the low contrast-enhancement, LIE-CT is only being used in a research setting and is currently not a clinically viable option to replace LGE-MRI.

## Comparison techniques

Table 1 shows an overview of the specifications of each technique described above.

**Table 1:** Specification overview of different CT techniques

	**Static CT-MPI, single energy**	**Static CT-MPI, dual-energy**	**Dynamic CT-MPI**	**LIE**
Average radiation dose	2–9 mSv (rest and stress)	4–16 mSv (rest and stress)	5–13 mSv (stress only)	2–5 mSv
Diagnostic accuracy				
**Sensitivity**	75–84%	89%	76–100%	98%
**Specificity**	78–95%	78%	74–100%	94%
Quantitative analysis	No	Yes	Yes	SECT no, DECT yes
Infarct and ischemia	Yes, with rest and stress	Yes, with rest and stress	Yes, stress only	No, infarct only

*CT-MPI* Computed tomography myocardial perfusion imaging, *LIE* Late iodine enhancement,* SECT* Single energy CT, *DECT* Dual energy CT

## Future perspectives

In this review, all current CT techniques for myocardial tissue characterization are discussed which are close to clinical implementation or have been a topic of patient-oriented research. However, there are some recent developed techniques that show promising preliminary results.

A promising hardware development is the development of photon-counting CT. Photon-counting CT uses a new energy-resolving x-ray detector that is able to count the number of incoming photons and measure photon energy and provides energy-resolved signals. Photon-counting CT has the potential to provide spectral information as an integral part of each scan, improved CNR for contrast-enhanced scans, and increased spatial resolution. This will result in new possibilities for the evaluation of coronary plaques or in-stent restenosis or the simultaneous evaluation of multiple contrast agents for different purposes [88].

One of the most promising software developments comes with the use of big data and the computational power to analyze this data. One of these early-stage developments is the use of texture analysis (TA), which refers to an objective and quantitative set of metrics for quantifying the texture of images. TA offers the potential of quantifying tissue characteristics that are disease specific and can be used to detect abnormalities that cannot be detected visually.

Thus, TA could have the potential to detect MI in cardiac CT images, which are potentially overlooked, especially when infarcts are small, readers have limited experience, or readers do not pay enough attention to the myocardium. TA has been used for other modalities such as MRI, and there are some recent studies showing that TA has a potential for CT analysis tool [78, 89, 90].

A closely related topic is radiomics which is defined as the extraction of large amounts of quantitative features from an image to create large data sets in which each abnormality is described by hundreds of parameters. From these large datasets, it is possible to establish correlations between the parameters and the clinical representation. Several research studies have shown the potential of radiomics in CT images [91–93]. Radiomics can be used to detect patterns from CT images that are able to distinguish between several myocardial. Often, machine learning algorithms are used to analyze these vast amounts of data and to find the often complex underlying relationships, bringing us to the next important development.

Due to developments of computer technology, artificial intelligence (AI) is now able to process large, real-time, high-resolution data sets. Given the complexity of cardiac CT imaging, AI offers the possibility to decrease evaluation time by automating measurements, reducing variability, and optimizing the efficiency of cardiac CT evaluation. The number of development-phase AI algorithms in cardiac CT research has been rapidly increasing. Currently, the majority of these AI applications are still focused on relatively straightforward but labor-intensive procedures such as calcium scoring, coronary plaque analysis, and cardiac fat quantification. However, new studies are merging that use large databases for risk assessment and prognostication [94]. Several studies have shown the potential of AI to aid in the analysis of MPI [95, 96].

## Data Availability

Not applicable

## Abbreviations

AI: Artificial intelligence
AIF: Arterial input function curve
CAD: Coronary artery disease
CCTA: Coronary computed tomography angiography
CT-MPI: Myocardial CT perfusion imaging
DECT: Dual-energy CT
DLCT: Dual-layer CT
DSCT: Dual-source CT
ECG: Electrocardiogram
LGE-MRI: Late gadolinium enhancement MRI
LIE-CT: Late iodine enhancement CT
MACE: Major adverse cardiovascular events
MBF: Myocardial blood flow
MBV: Myocardial blood volume
MDCT: Multi-detector CT
MPI: Myocardial perfusion imaging
PET: Positron emission tomography
SECT: Single energy CT
SPECT: Single-photon emission computed tomography
TA: Texture analysis
TAC: Tissue attenuation curves
VMI: Virtual mono-energetic images
